# Worldwide emergence of SARS-CoV-2 NB.1.8.1 sublineage with spike 420N and 478T dual substitution

**DOI:** 10.1080/22221751.2026.2755643

**Published:** 2026-10-26

**Authors:** Hoi-Wah Tsoi, Wan-Mui Chan, Allen Wing-Ho Chu, Cassie Yu-Sen Yip, Xiaojuan Zhang, Jonathan Daniel Ip, Yanling Luo, Hin Chu, Kwok-Yung Yuen, Kelvin Kai-Wang To

**Affiliations:** a State Key Laboratory of Emerging Infectious Diseases, Carol Yu Centre for Infection, Department of Microbiology, Li Ka Shing Faculty of Medicine, School of Clinical Medicine, the University of Hong Kong, Pokfulam, Hong Kong Special Administrative Region, People’s Republic of China; b Centre for Virology, Vaccinology and Therapeutics, Hong Kong Science and Technology Park, Shatin, Hong Kong Special Administrative Region, People’s Republic of China; c Department of Microbiology, Queen Mary Hospital, Pokfulam, Hong Kong Special Administrative Region, People’s Republic of China; d Pandemic Research Alliance Unit at the University of Hong Kong, Pokfulam, Hong Kong Special Administrative Region, People’s Republic of China; e Department of Infectious Disease and Microbiology, The University of Hong Kong-Shenzhen Hospital, Shenzhen, People’s Republic of China

**Keywords:** SARS-CoV-2, D420N, K478T or I478T, amino acid substitutions, evolution

## Abstract

**Background:**

After a year of quiescence, SARS-CoV-2 activity increased rapidly in Hong Kong since June 2026. We sought to characterize the genomic profile of SARS-CoV-2 driving this COVID-19 surge in Hong Kong and to examine the global dissemination of the identified circulating lineages.

**Methods:**

We performed SARS-CoV-2 whole-genome sequencing on archived respiratory specimens collected at Queen Mary Hospital, Hong Kong, between January 2025 and July 2026. Viral lineages were assigned via Nextclade. Global SARS-CoV-2 metadata downloaded from GISAID as of July 2026 were analysed to track global evolutionary trends.

**Results:**

Among 615 successfully sequenced strains, the XDV descendant NB.1.8.1 was the predominant lineage. Between April and July 2026, NB.1.8.1 descendants accounted for 95.6% (108/113) of sequenced strains, dominated by PQ.16.1.1 (81.5%). Spike substitutions 420N and 478T emerged rapidly. While 478T increased gradually throughout 2025, 420N emerged in April 2026 and reached 98.0% by June–July 2026. Strains harbouring 420N-478T dual substitution arose independently across distinct NB.1.8.1 lineages, accounting for 94.4% (102/108) of NB.1.8.1 descendants in Hong Kong during this period. Globally, the prevalence of NB.1.8.1 strains with 420N-478T dual substitution increased from <1% before April 2026 to 50.7% by mid-July 2026, with the highest prevalence observed in Southeast Asia and Australia.

**Conclusion:**

The 2026 COVID-19 surge in Hong Kong was driven by NB.1.8.1 descendants harbouring spike 420N and 478T substitutions. The rapid worldwide expansion of these dual-substitution lineages highlights their competitive growth advantage and underscores the need for continued global genomic surveillance.

## Introduction

Since emerging in late 2019, SARS-CoV-2 has evolved into different lineages, resulting in successive waves of infection. In Hong Kong, the first large SARS-CoV-2 outbreak occurred when the Omicron variant spread rapidly in early 2022, primarily driven by BA.2 [1,2]. Subsequent SARS-CoV-2 waves from 2022 to 2024 were characterized by BA.5, BA.2.3.20, XBB, EG.5.1 and JN.1 [3].

There is a noticeable shift in SARS-CoV-2 epidemiology in 2025 and 2026. Unlike the multiple discrete waves observed in preceding years, there was a single prolonged surge from spring to autumn in 2025, peaking in May, followed by a long period of quiescence from October 2025 to early spring 2026 [4]. However, SARS-CoV-2 activity rose sharply in June 2026. By mid-July 2026, SARS-CoV-2 was found in 10% of respiratory specimens tested at the Public Health Laboratory Services Branch in Hong Kong [4].

In this study, we conducted a longitudinal genomic surveillance using SARS-CoV-2 positive respiratory specimens collected during the 2025 and 2026 epidemic waves in Hong Kong. We demonstrate that JN.1-XDE recombinant lineage NB.1.8.1 predominated in both 2025 and 2026. Notably, the NB.1.8.1 in 2026 carried spike 420N and 478T dual substitution. Located in the receptor binding domain (RBD), these substitutions have been shown to confer immune escape or affect binding to the host receptor angiotensin-converting enzyme 2 (ACE2) [5–8]. Importantly, the dual substitution arose independently in different sublineages.

## Materials and methods

### Clinical specimens and nucleic acid extraction

Archived respiratory specimens collected between January 2025 and July 2026 and tested positive for SARS-CoV-2 by reverse transcriptase-polymerase chain reaction (RT-PCR) at Queen Mary Hospital in Hong Kong were retrieved. Total nucleic acid was extracted using the GeneRotex 96 Nucleic Acid Extractor using the Viral DNA and RNA Extraction Kit (T014H) (Tianlong).

### Viral genome sequencing and bioinformatics analysis

Whole viral genome sequencing directly from respiratory specimens was performed using the Oxford Nanopore MinION device (Oxford Nanopore Technologies, Oxford, United Kingdom) with the Midnight protocol as we described previously [9]. For bioinformatics analysis, consensus sequences were generated using wf-artic workflow (v1.0.0) via the EPI2ME Desktop application. A normalized value of 999999 and super accuracy mode for basecalling were applied as described previously [9,10]. The sequence MN908947.3 obtained from NCBI was used as the reference. The consensus sequences were verified manually using Integrative Genomics Viewer (IGV) v2.17.2 [11]. SARS-CoV-2 lineage was assigned using the online Nextclade tool (v3.21.2; https://clades.nextstrain.org; accessed on 31st July 2026). All sequences were deposited onto the Global Initiative on Sharing All Influenza Data (GISAID) database (Supplementary Table S1). A whole viral genome was considered successfully sequenced if at least 85% of the genome achieved a sequencing depth of ≥20x.

### Global SARS-CoV-2 sequence analysis

The analysis of global SARS-CoV-2 sequences was performed as we described previously [12]. We downloaded the SARS-CoV-2 sequences and metadata from GISAID on 29 July 2026. A SARS-CoV-2 sequence was included if it had been marked as “complete” and “betacoronavirus” in the metadata file and was directly obtained from a human clinical specimen without *in vitro* passage. A sequence was excluded if the collection date information was not complete, the sequence was originated from Hong Kong, both spike amino acid residue 420 and 478 could not be sequenced, or failed NextClade Cli analysis. The summary of the amino acid occurrence and monthly prevalence were calculated by an in-house python script using Python v3.9.11 with modules Pandas v3.0.0, and tqdm v4.67.1. The doubling time of strains with 478T only or 420N-478T dual substitution was determined as described previously [2].

### *In silico* analysis of ACE2 binding

The *in silico* analysis of ACE2 binding was performed using NextClade, which is based on the estimator from Starr et al. [13].

## Results

### Genomic surveillance in Hong Kong from January 2025 to July 2026

Between January 2025 and July 2026, we have sequenced a total of 772 strains collected in Queen Mary Hospital, in which 615 strains had at least 85% of the genome achieving a sequencing depth of ≥20x (Supplementary Table S2). NB.1.8.1, a descendant of XDV, was the predominant lineage during this period (Supplementary Figure S1). The median age of individuals with NB.1.8.1 is similar to those with non-NB.1.8.1 lineage (72 vs 68 years).

NB.1.8.1 descendants accounted for 95.6% (108/113) of strains successfully sequenced between 19th April and 6th July 2026, followed by XFG descendants (2.7%; 3/113) and BA.3.2 descendants (1.8%; 2/113) (Supplementary Table S3). Among the NB.1.8.1 descendants, PQ.16.1.1 (designated by the World Health Organization as a variant under monitoring on July 27, 2026 [14]) or its descendants, SV.1 and SV.2, was most predominant (81.5%; 88/108), followed by PQ.2 descendants (16.7%; 18/108), PQ.10 descendant (RH.1) (0.9%; 1/108), and PQ.17 descendant (RK.1) (0.9%; 1/108) (Supplementary Figure S2 and Supplementary Table S3).

The lineage defining amino acid substitutions include the spike protein D253G, N417T, and D420N for PQ.16.1, and K478T reversion for PQ.16.1.1 [15]. Notably, 420N is also lineage defining substitutions for RK.1, while 478T is also a lineage defining substitution for PQ.2.1 and PQ.17. Hence, we further analysed the prevalence of 420N and 478T among our strains. 478T exceeded 10% of all strains since July 2025 and peaked at 56.7% (17/30) in August 2025 (Figure 1(A)). In contrast, 420N was not found in 2025 and early 2026, but was first detected in April 2026. The proportion of strains with D420N substitution increased rapidly from 33.3% (2/6) in April 2026 to 98.0% (97/99) in June to July 2026, and almost all strains with 420N also carry 478T. 420N and/or 478T were only present among the NB.1.8.1 descendants, but not the XFG or BA.3.2 descendants (Supplementary Table S3). Among strains collected from April to July 2026, 420N, 478T and 420N-478T dual substitution were present in 97.2% (105/108), 97.2% (105/108) and 94.4% (102/108) of NB.1.8.1 descendants, respectively (Supplementary Table S3).

**Figure 1.**
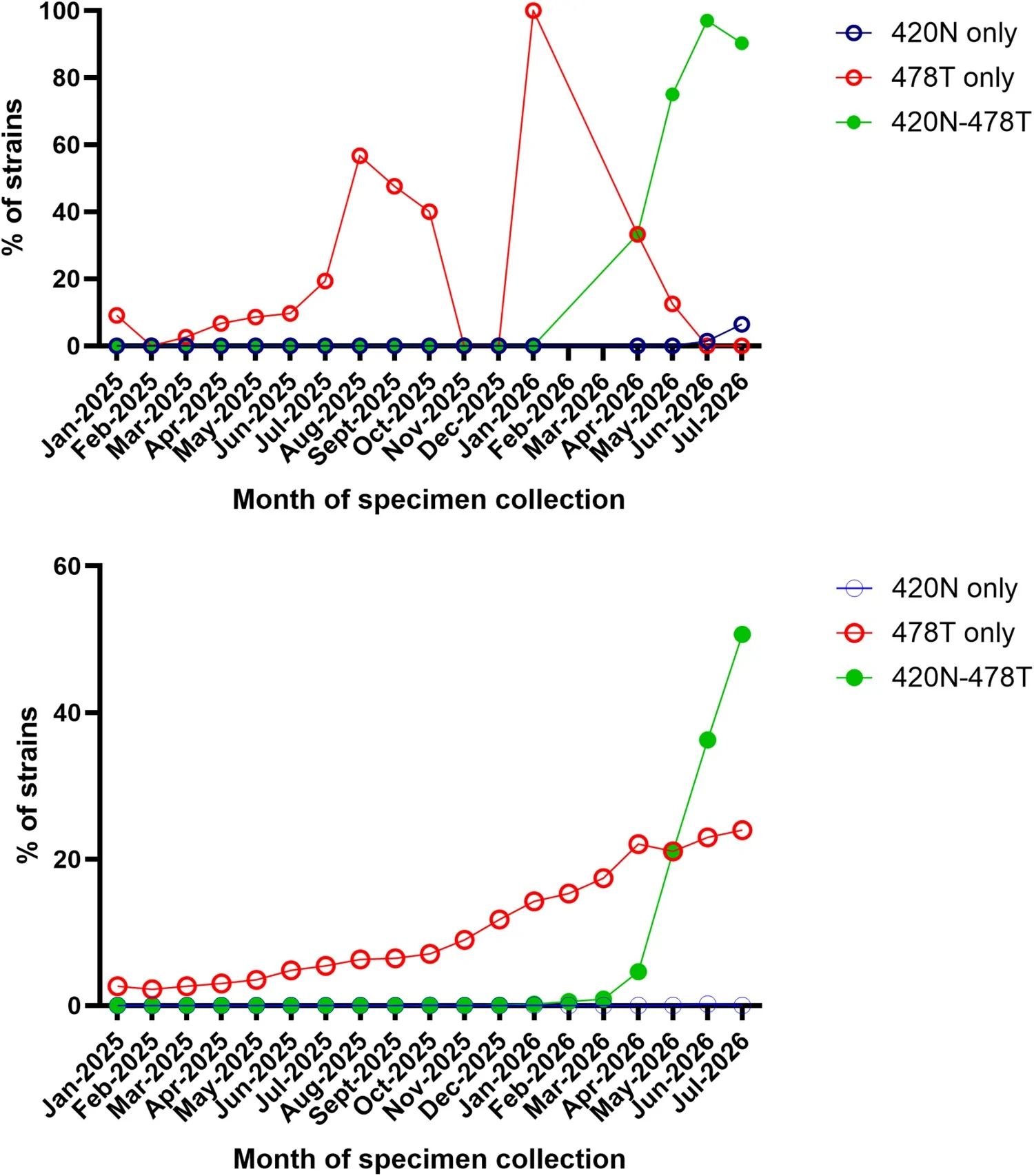
Prevalence of SARS-CoV-2 strains with spike 420N and/or 478T substitutions. (A) Our cohort. (B) Worldwide, excluding Hong Kong.

To determine if strains with 420N-478T dual substitution were also increasing worldwide, we analysed the data from GISAID, excluding data from Hong Kong. Our analysis showed a substantial increase in 420N-478T dual substitution worldwide in 2026. The prevalence of 420N-478T has increased from <1% in 2025 to 36.3% in June 2026 and 50.7% in July 2026 (Figure 1(B)). The doubling time of strains with 420N-478T dual substitution (1.21 months [95% confidence interval: 1.03–1.42]) was shorter than that of strains with 478T substitution only (5.46 months [95% confidence interval: 4.87–6.16]). Among the SARS-CoV-2 strains with 420N-478T dual substitution and collected between January 2025 and July 2026, 97.5% (1272/1304) were NB.1.8.1 descendants (Supplementary Table S4). The proportion of 420N-478T among NB.1.8.1 descendants varies between different regions. Among regions with at least 50 strains deposited in GISAID in 2026, 420N-478T dual substitution was most prevalent in Singapore (43.5%; 741/1704), followed by Thailand (34.6%; 27/78) and Taiwan (32.3%; 20/62) (Supplementary Table S5). Outside Asia, 420N-478T dual substitution was most prevalent in Australia (10.2%, 294/2879).

To determine whether 420N-478T dual substitution arose through convergent evolution or from a single common ancestor, we constructed a phylogenetic tree of all NB.1.8.1 strains from Queen Mary Hospital collected between January 2025 and July 2026. The phylogenetic tree showed that NB.1.8.1 strains harbouring 420N-478T dual substitution emerged independently across distinct lineages instead of descending from a single common ancestor (Supplementary Figure S3).

To investigate whether the rapid increase in the prevalence of 420N-478T is related to ACE2 binding, we evaluated the ACE2 binding using NextClade, which is based on the work by Starr et al. [13]. Compared to the BA.2.86 baseline, the ACE2 binding affinities of the PQ.16-420D-478I, PQ.16-420D-I478T, and PQ.16-D420N-I478T are −2.25882, −2.2775 and −2.31451 Δ-log_10_(*K_D_*), respectively, suggesting that 420N-478T dual substitution confers a slightly lower binding affinity to ACE2.

## Discussion

In this genomic surveillance study, we identified the rapid emergence of NB.1.8.1 descendants carrying spike 420N-478T dual substitution during the 2026 summer COVID-19 season in Hong Kong. Notably, 420N-478T dual substitution has arisen independently across multiple genetically divergent NB.1.8.1 sublineages, including PQ.16.1.1, PQ.2.1.11, and RK.1, suggesting convergent evolution. NB.1.8.1 descendants with 420N-478T substitutions also increased substantially worldwide, especially in Southeast Asia and Australia.

The acquisition of the D420N substitution within the RBD appears to be a key molecular determinant of this ongoing SARS-CoV-2 surge. Witte *et al* found that the D420N substitution emerged during the passage of SARS-CoV-2 BA.2 variant with monoclonal neutralizing antibody [5]. Synthetic virus with D420N substitution showed a substantial reduction in the neutralizing activity of class 1/2 or class 1/4 antibodies. Furthermore, deep mutational scanning by Dadonaite *et al* highlighted D420N as a primary escape mutation against polyclonal serum antibodies derived from individuals vaccinated and infected during the XBB era [6]. Recently, He *et al* found that both PQ.16.1.1 and RK.1, both with D420N as clade defining substitutions, evade some class 1 monoclonal antibodies [16]. Notably, the impact of D420N is strain dependent. D420N affects the neutralizing activity of C3095 (a class 1/2 mAb) for BA.1 or BA.2 but not the ancestral strain [5].

Almost all strains with spike 420N also carry 478T. While T478K substitution was the hallmark of the Delta variant, the initial NB.1.8.1 strains possessed 478I. Previous studies showed that different substitutions at residue 478 can affect ACE2 binding. While T478K enhances the binding, T478I does not significantly affect ACE2 binding [7,8]. Strains with 478T reversion rapidly increased since July 2025 to about 50%. However, strains with 478T without 420N did not further expand in 2025. Instead, the expansion of NB.1.8.1 only occurred in 2026 when 478T was accompanied by 420N, suggesting that the advantage in transmission or human infection of 420N-478T dual substitution over those with single 478T substitution. Our *in silico* results showed that 420N-478T dual substitution had a slightly lower binding affinity to ACE2 receptor. This result concurs with a recent study which showed that PQ.16.1.1 has lower binding affinity to the ACE2 receptor than NB.1.8.1 using a pseudovirus binding assay [16]. Further studies are required to confirm whether the 420N-478T dual substitution is responsible for the change in ACE2 binding affinity and whether the change in binding affinity affect viral fitness and transmission in humans.

There are several limitations in this study. First, we only included clinical specimens from hospital patients, and therefore the lineages from milder cases were excluded. Second, we only included clinical specimens from a single hospital, which may not be generalizable to the entire city. Third, there is substantial variation in the number of sequences uploaded to GISAID between countries or regions. Estimates of variant prevalence would be less accurate for places with fewer sequences. Furthermore, the estimated global prevalence may be disproportionately influenced by places with a higher number of sequences.

In summary, the 2026 COVID-19 surge in Hong Kong was predominated by NB.1.8.1 descendants with 420N-478T dual substitution, which arose independently in different lineages. Although strains with 420N-478T substitutions are spreading rapidly, there is currently no epidemiological evidence to suggest that these strains cause more severe disease. Further studies are required to determine how the dual substitution has competitive advantages over strains with single substitution, especially for immune escape and receptor binding.

## Supplementary Material

3_Supple_Table_S6_20260915.pdf

3_Supple_Table_S1.xlsx

3_Supple_Table_S2_S5_Fig_S1_S2_20260916_1654_CLEAN.docx
