# Supplementary material for: Worldwide emergence of SARS-CoV-2 NB.1.8.1 sublineage with spike 420N and 478T dual substitution
Source: Emerg Microbes Infect. 2026 Oct 26;15(1):2755643. doi: 10.1080/22221751.2026.2755643 (PMC13757857; doi:10.1080/22221751.2026.2755643)
Supplement: Supplementary file 1 [file TEMI_A_2755643_SM9694.pdf]

Supplementary Appendix

All genome sequences and associated metadata supporting the findings of this study can be accessed through the persistent digital object identifier https://doi.org/10.55876/gis8.260802ge

In addition to the minted DOI, GISAID also communicates the aggregation of GISAID accession numbers (EPI_ISL_IDs) through the corresponding EPI_SET_260802ge identifier to facilitate both, the acknowledgment of all data contributors and the direct retrieval of the underlying data from GISAID used in this study.

hCoV-19 Virus Data Summary

GISAID Identifier	Digital Object Identifier	Number of individual viruses	Data Collection range	Number of countries/territories
EPI_SET_260802ge	https://doi.org/10.55876/gis8.260802ge	1,125,735	2023-09-01 to 2026-07-17	137
