# Supplementary material for: Worldwide emergence of SARS-CoV-2 NB.1.8.1 sublineage with spike 420N and 478T dual substitution
Source: Emerg Microbes Infect. 2026 Oct 26;15(1):2755643. doi: 10.1080/22221751.2026.2755643 (PMC13757857; doi:10.1080/22221751.2026.2755643)
Supplement: Supplementary file 2 [file TEMI_A_2755643_SM9692.docx]

**Supplementary Table S2**. Number of SARS-CoV-2 strains with successful whole genome sequenced from January 2025 to July 2026.

| **Month of collection** | **Number of specimens** |
| --- | --- |
| Jan-2025 | 11 |
| Feb-2025 | 11 |
| Mar-2025 | 38 |
| Apr-2025 | 150 |
| May-2025 | 128 |
| Jun-2025 | 72 |
| Jul-2025 | 31 |
| Aug-2025 | 30 |
| Sept-2025 | 21 |
| Oct-2025 | 5 |
| Nov-2025 | 2 |
| Dec-2025 | 1 |
| Jan-2026 | 2 |
| Feb-2026 | 0 |
| Mar-2026 | 0 |
| Apr-2026 | 6 |
| May-2026 | 8 |
| Jun-2026 | 68 |
| Jul-2026^a^ | 31 |
| Total | 615 |

^a^ Up to 6^th^ July 2026

**Supplementary Table S3**. SARS-CoV-2 lineages between April and July 2026 in our cohort

| **Lineage** | **Sublineage** | **No. of strains (%)** | | | |
| --- | --- | --- | --- | --- | --- |
|  |  | Total | 420N^d^ | 478T^e^ | Both 420N and 478T |
| **NB.1.8.1 descendants** | PQ.16.1.1 | 6 | 6  (100) | 6  (100) | 6  (100) |
|  | SV.1^a^ | 37 | 37  (100) | 37  (100) | 37  (100) |
|  | SV.1.1^a^ | 9 | 9  (100) | 9  (100) | 9  (100) |
|  | SV.2^a^ | 35 | 35  (100) | 35  (100) | 35  (100) |
|  | SV.2.1^a^ | 1 | 1  (100) | 1  (100) | 1  (100) |
|  | PQ.2.1.11 | 14 | 13  (92.9) | 14  (100) | 13 (92.9) |
|  | PQ.2.1.7 | 1 | 0  (0) | 1  (100) | 0  (0) |
|  | PQ.2.8.1 | 3 | 3  (100) | 0  (0) | 0  (0) |
|  | RH.1^b^ | 1 | 0  (0) | 1  (100) | 0  (0) |
|  | RK.1^c^ | 1 | 1  (100) | 1  (100) | 1  (100) |
|  | **Subtotal** | 108 | 105  (97.2) | 105 (97.2) | 102 (94.4) |
| **XFG descendants** | XFG.5.2.8 | 1 | 0  (0) | 0  (0) | 0  (0) |
|  | XFG.6 | 1 | 0  (0) | 0  (0) | 0  (0) |
|  | XFG.6.13 | 1 | 0  (0) | 0  (0) | 0  (0) |
|  | **Subtotal** | 3 | 0  (0) | 0  (0) | 0  (0) |
| **BA.3.2 descendants** | RE.1.1.5 | 1 | 0  (0) | 0  (0) | 0  (0) |
|  | SQ.3 | 1 | 0  (0) | 0  (0) | 0  (0) |
|  | **Subtotal** | 2 | 0  (0) | 0  (0) | 0  (0) |
| **Total** |  | 113 | 105  (92.9) | 105  (92.9) | 102  (90.3) |

^a^ Descendant of PQ.16.1.1

^b^ Descendant of PQ.10.1.4

^c^ Descendant of PQ.17.7.2

^d^ Lineage defining amino acid substitution for PQ.16.1, RK.1

^e^ Lineage defining amino acid substitution for PQ.16.1.1, PQ.2.1 and PQ.17

**Supplementary Table S4**. Lineages of global SARS-CoV-2 strains with spike 420N-478T dual substitution collected between January 2025 and July 2026.

|  | **No. of sequences**  (n=1304) |
| --- | --- |
| **NB.1.8.1 descendants** | 1272 |
| NB.1.8.1 | 55 |
| PQ.10 | 1 |
| PQ.14 | 6 |
| PQ.16 | 649 |
| PQ.17 | 275 |
| PQ.2 | 1 |
| PQ.22 | 7 |
| PQ.23 | 1 |
| PQ.43 | 220 |
| PQ.5 | 4 |
| PQ.50.1 | 52 |
| PQ.6 | 1 |
| **Others** | 32 |

**Supplementary Table S5**. Proportion of spike 420N-478T substitution in different regions among SARS-CoV-2 strains collected in 2026^a^.

| **Regions** | **% of sequences with spike 420N-478T available at GISAID** |
| --- | --- |
| Singapore | 43.5% (741/1704) |
| Thailand | 34.6% (27/78) |
| Taiwan | 32.3% (20/62) |
| Malaysia | 12.6% (12/95) |
| Australia | 10.2% (294/2879) |
| Sweden | 4.8% (22/462) |
| China | 2.9% (5/170) |
| Germany | 2.9% (15/518) |
| Ireland | 2.1% (3/143) |
| Norway | 1.7% (2/121) |
| Denmark | 1.3% (3/237) |
| Spain | 0.7% (3/410) |
| Canada | 0.7% (52/7630) |
| USA | 0.6% (67/11131) |
| France | 0.6% (2/356) |
| Netherlands | 0.5% (1/182) |
| Japan | 0.5% (2/436) |
| Russia | 0.2% (1/659) |

^a^ Only included regions with at least 50 strains uploaded to GISAID in 2026, and excluded strains from Hong Kong

**Supplementary Figure S1**. The trend of SARS-CoV-2 lineages from January 2025 to July 2026 in our cohort. Data from July 2026 is from July 1 to 6 only.

**Supplementary Figure S2.** The proportion of NB.1.8.1 sublineages from April 19 to July 6, 2026, in our cohort.

**Supplementary Figure S3.** Whole genome phylogenetic analysis of SARS-CoV-2 NB.1.8.1 genomes between January 2025 and July 2026 from Queen Mary Hospital in Hong Kong. The whole-genome phylogenetic tree was constructed using the Nextclade tool (version 3.23.0). The tree was visualized and exported using the online tool [www.auspice.us](http://www.auspice.us). The orange nodes represent strains with spike 420N-478T dual substitution.
